# Supplementary material for: Association between prehospital time and outcome of trauma patients in 4 Asian countries: A cross-national, multicenter cohort study
Source: PLoS Med. 2020 Oct 6;17(10):e1003360. doi: 10.1371/journal.pmed.1003360 (PMC7537901; doi:10.1371/journal.pmed.1003360)

**S1 Text**

**S1 Text Table A.** The originally submitted study proposal and its analysis plan of current study.

**S1 Text Table B.** Numbers (%) of valid prehospital timing record in each country

**S1 Text Table C.** Comparison of prehospital time in patients included in the study of 30-day mortality (n=24365)

**S1 Text Table D.** Multivariable linear regression analysis evaluating factors influencing total hospital time

**S1 Text Table E.** Comparison of demographic characteristics in different country

**S1 Text Table F.** Comparison of included sample and excluded sample due to missing data

**S1 Text Fig A.** Using ROC curve and Youden Index (YI) to determine cutoff value of Total prehospital time (min) for predicting poor functional outcome. TPT≥50 min is the best cutoff value to predict poor functional outcome with the maximum YI (Sensitivity + specificity)

**S1 Text Fig B.** Comparison of mean predicted and observed outcome rates of TPT per 10 minutes (%)

**S1 Text Table A.** The originally submitted study proposal and its analysis plan of current study.

| 1. **BASIC INFORMATION** | |
| --- | --- |
| **Name:** **Wen-Chu, Chiang** | **Email: drchiang.tw@gmail.com** |
| **Country: TAIWAN** | **Institution: National Taiwan University Hospital** |
| 1. **TYPE OF REQUEST** | |
| ■ New study proposal (initial submission)  □ Secondary analysis  □ Explanatory analysis | |
| 1. **FIELD OF STUDY (select one)** | |
| □ 1 Trauma epidemiology and prevention  ■ 2 EMS trauma research  ■ 3 ED trauma research  □ 4 Hospital trauma research  □ 5 Trauma system monitoring research | |
| 1. **STUDY TITLE** | |
| **Association between timeliness and outcome of patients with major trauma** | |
| 1. **ABSTRACT OF STUDY PROPOSAL** | |
| **Describe the study under the given headings below.** | |
| **Background**   1. Treatment of patients with major trauma emphasizes the “load-and-go” principle to reduce the time between occurrence of injury and definite care in destination hospital. 2. Concept of “golden one hour” has been proposed for over 40 years, without much evidence to support. 3. Optimal timeliness of EMS scene time and ED stay time has not been examined among Asian population. | |
| **Objectives/Hypotheses (P/E/O style in observational study)**  In PATOS data, (P) patients with major trauma (ISS>15) would have better outcomes of survival to discharge (O1) and long-term functional status (O2) if the timeliness from injury occurrence to definite care (operation or angiography) is shorter (E). | |
| **Methodology** (To include study setting; population of interest; inclusion and exclusion criteria; sample size; exposure or intervention variable; control group; outcome variable; and types of statistical analysis, etc).   1. Study setting: PATOS data 2. Population of interest: patients with major trauma (ISS>15) 3. Inclusion and exclusion criteria: Excluded those without records of ISS or outcomes 4. Sample size: As an observational study, we should use all the available cases as possible 5. Exposure or intervention : timeliness of (1) response tine (2) scene time (3) transport time (4) ED entrance time to definite care (operation or angiography) 6. Control group: Non-applicable in observational study 7. Outcome variables: survival to discharge (O1) and long-term functional status (O2) 8. Statistical analysis: logistic regression and general linear model regression | |
| **Significance of the study** ( e.g. a brief description on how the study can improve current systems, its benefit to patients, and how it can be implemented)   1. Provide evidence for the old dogma “golden one hour”, or propose another optimal timeliness for prehosptal care and ED care 2. Evidence raised from Asian population may change the local trauma care policy in the future | |

| **S1 Text Table B.** Numbers (%) of valid prehospital timing record in each country | | | | | | |
| --- | --- | --- | --- | --- | --- | --- |
| Country | Total | Injury | EMS call received | EMS arrival at scene | Leave scene | Arrive at ED |
| CN | 34 | 34 (100.0) | 2 (5.9) | 2 (5.9) | 2 (5.9) | 34 (100.0) |
| IN | 173 | 152 (87.9) | 42 (24.3) | 41 (23.7) | 41 (23.7) | 153 (88.4) |
| JP | 919 | 766 (83.4) | 919 (100.0) | 919 (100.0) | 919 (100.0) | 918 (99.9) |
| KR | 31548 | 30960 (98.1) | 621 (2.0) | 30511 (96.7) | 2130 (6.8) | 31542 (100.0) |
| MY | 12454 | 11326 (90.9) | 11858 (95.2) | 11785 (94.6) | 11745 (94.3) | 12294 (98.7) |
| PH | 17 | 17 (100.0) | 2 (11.8) | 2 (11.8) | 1 (5.9) | 17 (100.0) |
| TH | 40 | 39 (97.5) | 32 (80.0) | 32 (80.0) | 32 (80.0) | 40 (100.0) |
| TW | 2845 | 2845 (100.0) | 2845 (100.0) | 2841 (99.9) | 2840 (99.8) | 2818 (99.1) |
| VN | 318 | 234 (73.6) | 69 (21.7) | 71 (22.3) | 68 (21.4) | 318 (100.0) |
| Overall | 48348 | 46373 (95.9) | 16390 (33.9) | 46204 (95.6) | 17778 (36.8) | 48134 (99.6) |
| CN=China, ED=emergency department, IN=India, JP=Japan, KR=Korea, MY=Malaysia,  PH=Philippines, TH=Thailand, TW=Taiwan, VM=Vietnam | | | | | | |

| **S1 Text Table C.** Comparison of prehospital time in patients included in the study of 30-day mortality (n=24365) | | | | | | |
| --- | --- | --- | --- | --- | --- | --- |
|  | TPT, min | p | RT, min | p | SH, min | p |
| **Age, year** |  | 0.128 |  | <0.001 |  | <0.001 |
| <60 | 46.0 (29.0) |  | 21.0 (27.0) |  | 21.0 (14.0) |  |
| ≥60 | 47.0 (27.0) |  | 20.0 (27.0) |  | 22.0 (12.0) |  |
| **Sex** |  | 0.008 |  | 0.002 |  | 0.349 |
| Male | 47.0 (28.0) |  | 21.0 (27.0) |  | 21.0 (13.0) |  |
| Female | 46.0 (29.0) |  | 20.0 (26.0) |  | 21.0 (13.0) |  |
| **MOI** |  | <0.001 |  | 0.180 |  | <0.001 |
| Non-penetrating | 47.0 (28.0) |  | 20.0 (27.0) |  | 21.0 (13.0) |  |
| Penetrating | 43.0 (30.0) |  | 20.0 (25.0) |  | 19.0 (13.0) |  |
| **TOI** |  | <0.001 |  | <0.001 |  | <0.001 |
| No TBI | 49.0 (29.0) |  | 22.0 (27.0) |  | 21.0 (13.0) |  |
| Mixed TBI | 44.0 (29.0) |  | 19.0 (23.0) |  | 21.0 (14.0) |  |
| Isolated TBI | 42.0 (30.0) |  | 18.0 (24.0) |  | 20.0 (12.0) |  |
| **ISS** |  | 0.409 |  | 0.006 |  | <0.001 |
| <16 | 47.0 (28.0) |  | 20.0 (27.0) |  | 21.0 (13.0) |  |
| ≥16 | 45.0 (26.0) |  | 20.0 (16.0) |  | 23.0 (14.0) |  |
| **RTS** |  | <0.001 |  | <0.001 |  | 0.015 |
| ≥7 | 47.0 (28.0) |  | 21.0 (27.0) |  | 21.0 (13.0) |  |
| <7 | 41.0 (28.0) |  | 19.0 (15.0) |  | 20.0 (13.0) |  |
| **Prehospital rescue airway†** |  | 0.352 |  | 0.840 |  | 0.039 |
| No | 47.0 (28.0) |  | 20.0 (27.0) |  | 21.0 (13.0) |  |
| Yes | 52.0 (45.8) |  | 24.0 (30.3) |  | 27.0 (18.0) |  |
| **Prehospital IV/IO access** |  | <0.001 |  | <0.001 |  | <0.001 |
| No | 46.0 (28.0) |  | 20.0 (26.0) |  | 21.0 (13.0) |  |
| Yes | 57.0 (36.0) |  | 28.0 (27.0) |  | 25.0 (15.0) |  |
| **Status after 30 days** |  | 0.006 |  | 0.020 |  | 0.754 |
| Survival | 47.0 (28.0) |  | 20.0 (27.0) |  | 21.0 (13.0) |  |
| Mortality | 41.0 (25.3) |  | 19.0 (9.8) |  | 20.0 (12.8) |  |
| **Functional outcome at discharge*** |  | <0.001 |  | <0.001 |  | <0.001 |
| Favorable | 48.0 (30.0) |  | 20.0 (29.0) |  | 21.0 (14.0) |  |
| Poor | 56.0 (37.0) |  | 26.0 (31.0) |  | 24.0 (16.0) |  |
| **Overall** | 47.0 (28.0) | NA | 20.0 (27.0) | NA | 21.0 (13.0) | NA |
| ETT=endotracheal tube, IO=intraosseous line, ISS=injury severity score, IV=intravenous line, MOI=mechanism of injury, NA=not available, p=p value comparing survival between mortality, P=p value between subgroup, RT=response time, SH=scene to hospital time, TBI=traumatic brain injury, TOI=type of injury, TPT=total prehospital time, TT=transport time All data was reported as median (IQR) * *n* = 21886 † Rescue airway: include prehospital supraglottic airway and endotracheal tube insertion | | | | | | |

| **S1 Text Table D.** Multivariable linear regression analysis evaluating factors influencing total hospital time | | |
| --- | --- | --- |
| Variables | β(SE) | *p* |
| **Age** | 0.03 (0.01) | <0.001 |
| **Male** | 0.01 (0.38) | 0.027 |
| **Penetrating injury** | -0.03 (0.90) | <0.001 |
| **Traumatic brain injury** | -0.08 (0.39) | <0.001 |
| **ISS≥16** | 0.01 (0.76) | 0.349 |
| **RTS < 7** | -0.04 (0.89) | <0.001 |
| **Prehospital rescue airway⃰** | 0.01 (6.59) | 0.158 |
| **Prehospital IV/IO access** | 0.08 (0.83) | <0.001 |
| IO=intraosseous line, ISS=injury severity score, IV=intravenous line *Rescue airway: include prehospital supraglottic airway and endotracheal tube insertion | | |

| **S1 Text Table E.** Comparison of demographic characteristics in different country | | | | | |
| --- | --- | --- | --- | --- | --- |
| Variables | JP | KR | MY | TW | *p* |
| Age | 47.0 (43.0) | 51.0 (35.0) | 28.0 (23.0) | 53.0 (37.0) | <0.001 |
| Male | 399 (61.9) | 9711 (58.9) | 4240 (79.1) | 1148 (60.9) | <0.001 |
| TBI | 257 (39.8) | 5778 (35.1) | 1503 (28.1) | 654 (34.7) | <0.001 |
| ISS≥16 | 34 (5.3) | 632 (3.8) | 506 (9.4) | 466 (24.7) | <0.001 |
| RTS<7 | 21 (3.3) | 528 (3.2) | 259 (4.8) | 365 (19.4) | <0.001 |
| RT (min) | 22.0 (21.0) | 17.0 (28.0) | 30.0 (26.0) | 20.0 (3.0) | <0.001 |
| SH (min) | 30.0 (13.0) | 20.0 (13.0) | 24.0 (15.0) | 21.0 (9.0) | <0.001 |
| TPT (min) | 55.0 (28.0) | 43.0 (30.0) | 58.0 (34.0) | 41.0 (22.0) | <0.001 |
| 30-day mortality | 1 (0.2) | 105 (0.6) | 56 (1.0) | 118 (6.3) | <0.001 |
| Poor functional outcome | 22 (4.0) | 902 (5.5) | 563 (11.4) | NA⃰ | <0.001 |
| ISS=Injury severity score, JP=Japan, KR=Korea, MY=Malaysia, NA=not available,  RT=response time, RTS=revised trauma score, SH=scene to hospital time,  TBI=traumatic brain injury, TPT=total prehospital time, TW=Taiwan *There was no record of functional outcome in Taiwan | | | | | |

| **S1 Text Table F.** Comparison of included sample and excluded sample due to missing data | | | | | |
| --- | --- | --- | --- | --- | --- |
|  | numbers of missing record (%) | Total (n=48348) | included sample (n=24365) | excluded sample (n=23983) | p |
| Age | 152 (0.3) | 43.0 (39.0) | 45 (37) | 41 (39) | <0.001 |
| Sex | 62 (0.1) | 30629 (63.4) | 15498 (63.6) | 15131 (63.3) | 0.420 |
| ISS ≥ 16 | 7419 (15.3) | 38087 (93.1) | 1638 (6.7) | 1204 (7.3) | 0.033 |
| RTS < 7 | 7355 (15.2) | 2463 (6.0) | 1174 (4.8) | 1290 (7.8) | <0.001 |
| Response time | 11529 (23.8) | 20.0 (26.0) | 20.0 (27.0) | 20.0 (24.0) | 0.043 |
| Scene to hospital time | 4419 (9.1) | 22.0 (14.0) | 21.0 (13.0) | 23.0 (16.0) | <0.001 |
| Total prehospital time | 7555 (15.6) | 46.0 (35.0) | 47.0 (28.0) | 45.0 (47.0) | 0.590 |
| Mortality in 30 days | 0 (0%) | 1324 (2.7) | 280 (1.1) | 1044 (4.4) | <0.001 |
| Poor functional outcome at discharge | 4882 (10.1) | 3822 (8.8) | 1487 (6.8) | 2335 (10.8) | <0.001 |
| ISS=injury severity score, RTS=revised trauma score Dichotomous and categorical variables were reported as absolute sample size (percentages), whereas continuous variables were reported as median (IQR) | | | | | |

**S1 Text Fig A.** Using ROC curve and Youden Index (YI) to determine cutoff value of Total prehospital time (min) for predicting poor functional outcome. TPT≥50 min is the best cutoff value to predict poor functional outcome with the maximum YI (Sensitivity + specificity)


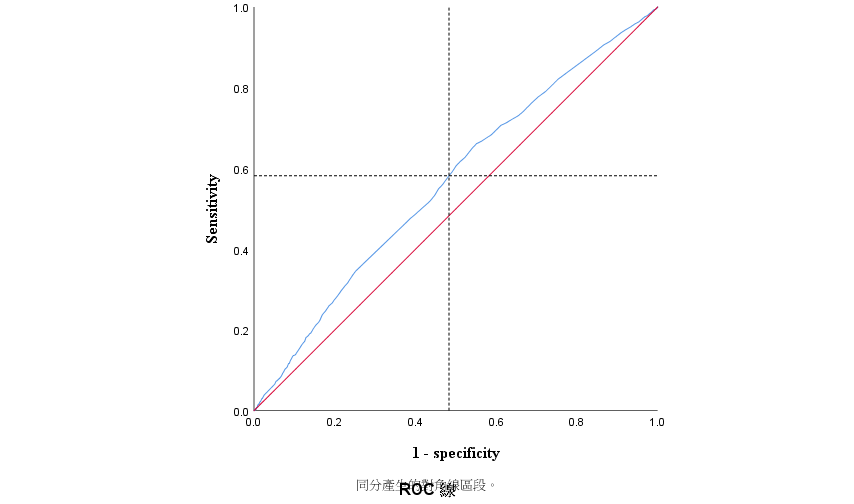


**S1 Text Fig B.** Comparison of mean predicted and observed outcome rates of TPT per 10 minutes (%)


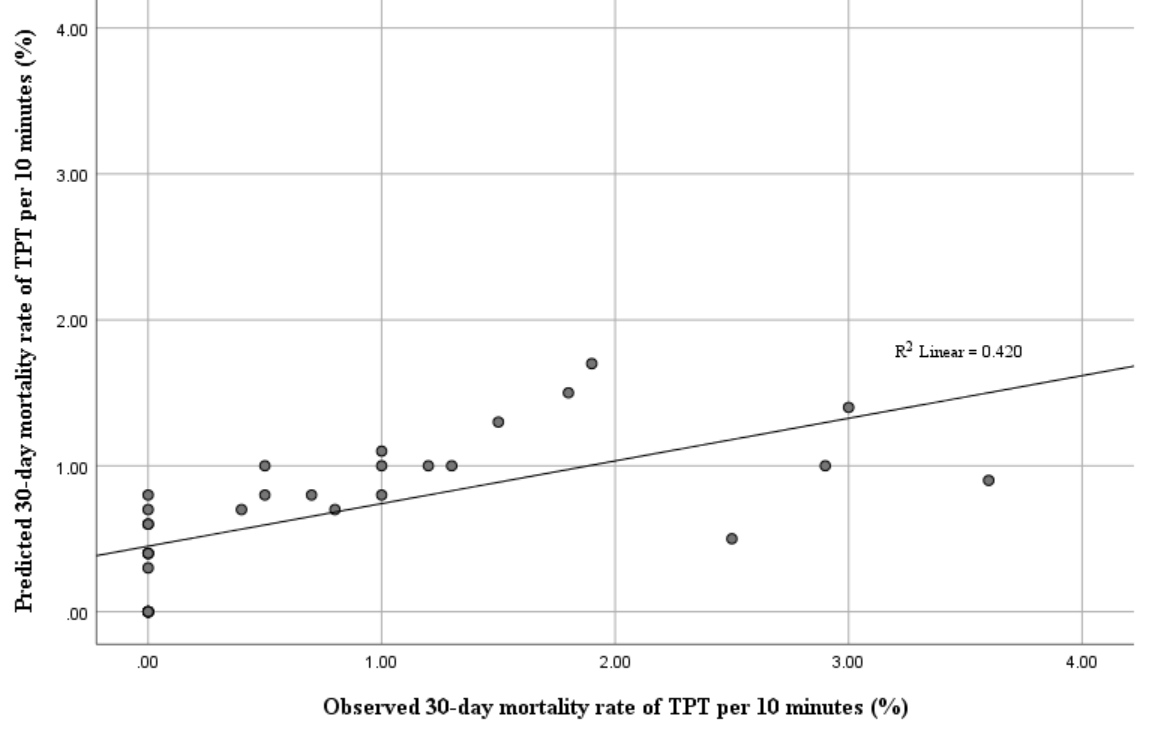

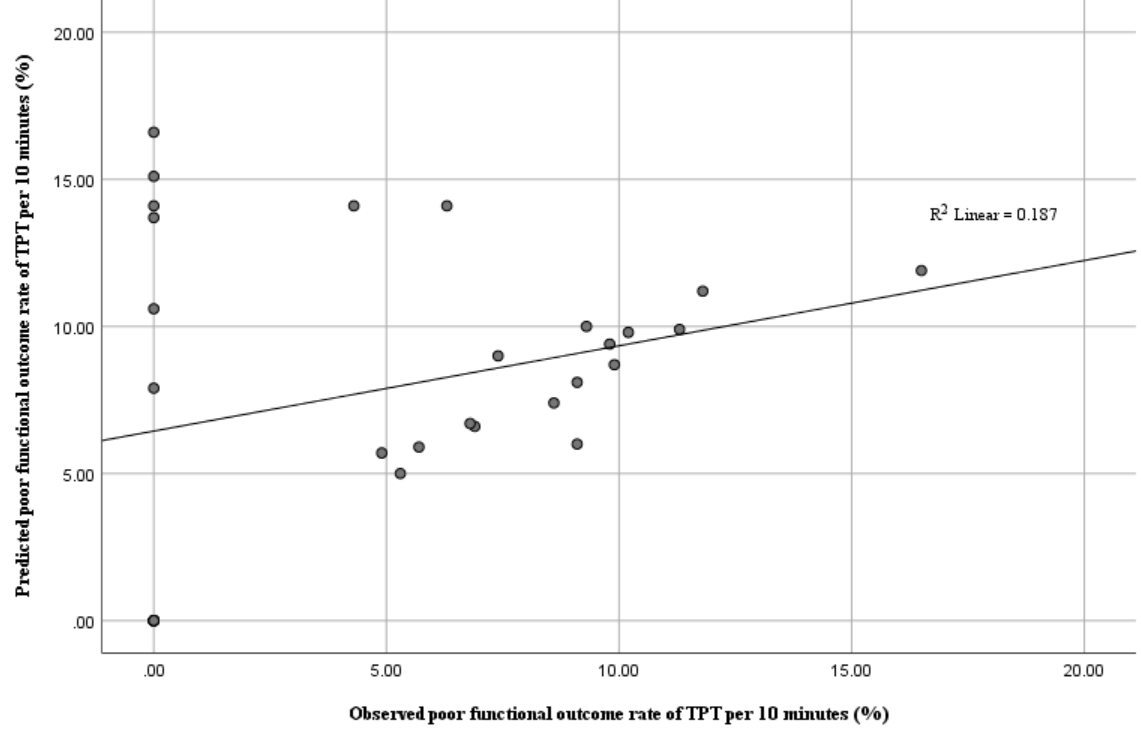

Supplement: S1 Text — Tables A–F and Figs A and B. (DOCX) [file pmed.1003360.s002.docx]
