# Supplementary material for: Association between prehospital time and outcome of trauma patients in 4 Asian countries: A cross-national, multicenter cohort study
Source: PLoS Med. 2020 Oct 6;17(10):e1003360. doi: 10.1371/journal.pmed.1003360 (PMC7537901; doi:10.1371/journal.pmed.1003360)
Supplement: Attachment — Submitted filename: Response To Reviewers.docx [file pmed.1003360.s003.docx]

Response To Reviewers

Dear editors,

We have completed the submission by providing the additional information.

This manuscript adheres to the appropriate reporting guidelines of the STROBE statement and community standards for data availability. The STROBE checklist used in conjunction with this article (available on the Web sites of PLoS Medicine at <http://www.plosmedicine.org/>) has been included in the submission files.

This manuscript has not been published or presented elsewhere in part or in entirety and is not under consideration by another journal. The study design was approved by the appropriate ethics review board. We have read and understood your journal’s policies, and we believe that neither the manuscript nor the study violates any of these. There are no conflicts of interest to declare.

Thank you for your consideration. I look forward to hearing from you.

Sincerely,

Wen-Chu Chiang

[Affiliation] Department of Emergency Medicine, National Taiwan University Hospital, Yun-Lin Branch, Douliu City, Taiwan

[Postal address] No. 7, Chung-Shan S. Rd, Taipei, Taiwan

[Phone number] +886-2-23123456 ext. 62831

[Fax number] +886-2-23223150

E-mail address: [drchiang.tw@gmail.com](mailto:drchiang.tw@gmail.com)
