# Supplementary material for: Association between prehospital time and outcome of trauma patients in 4 Asian countries: A cross-national, multicenter cohort study
Source: PLoS Med. 2020 Oct 6;17(10):e1003360. doi: 10.1371/journal.pmed.1003360 (PMC7537901; doi:10.1371/journal.pmed.1003360)
Supplement: Attachment — Submitted filename: PMEDICINE-D-20-02427R1 Author Responses_final_0709CWC.docx [file pmed.1003360.s004.docx]

**Response to Reviewers’ Comments**

Manuscript Number: PMEDICINE-D-20-02427R1

Title: **Association between prehospital time and outcome of trauma patients in Asia: A cross-national, multicenter, retrospective cohort study**

*(Previously entitled:* *Association between prehospital time and outcome of trauma patients in Asia: Does the “golden hour” matter?)*

PMEDICINE-D-20-02427R1 Review Opinions

Dear Dr. WEN-CHU,

Thank you very much for submitting your manuscript "Association between prehospital time and outcome of trauma patients in Asia: Does the “golden hour” matter?" (PMEDICINE-D-20-02427R1) for consideration at PLOS Medicine.

Your paper was evaluated by a senior editor and discussed among all the editors here. It was also discussed with an academic editor with relevant expertise, and sent to independent reviewers, including a statistical reviewer. The reviews are appended at the bottom of this email and any accompanying reviewer attachments can be seen via the link below:

In light of these reviews, I am afraid that we will not be able to accept the manuscript for publication in the journal in its current form, but we would like to consider a revised version that addresses the reviewers' and editors' comments. Obviously we cannot make any decision about publication until we have seen the revised manuscript and your response, and we plan to seek re-review by one or more of the reviewers.

In revising the manuscript for further consideration, your revisions should address the specific points made by each reviewer and the editors. Please also check the guidelines for revised papers at http://journals.plos.org/plosmedicine/s/revising-your-manuscript for any that apply to your paper. In your rebuttal letter you should indicate your response to the reviewers' and editors' comments, the changes you have made in the manuscript, and include either an excerpt of the revised text or the location (eg: page and line number) where each change can be found. Please submit a clean version of the paper as the main article file; a version with changes marked should be uploaded as a marked up manuscript.

In addition, we request that you upload any figures associated with your paper as individual TIF or EPS files with 300dpi resolution at resubmission; please read our figure guidelines for more information on our requirements: http://journals.plos.org/plosmedicine/s/figures. While revising your submission, please upload your figure files to the PACE digital diagnostic tool, https://pacev2.apexcovantage.com/. PACE helps ensure that figures meet PLOS requirements. To use PACE, you must first register as a user. Then, login and navigate to the UPLOAD tab, where you will find detailed instructions on how to use the tool. If you encounter any issues or have any questions when using PACE, please email us at PLOSMedicine@plos.org.

**Response from the authors:**

Thank you. We have uploaded the figures in our article to the PACE and adjusted the figures to meet PLOS requirements. We will re-upload these files.

**Changes made (Please refer to the marked version):**

Figures 1-4 were revised.

We expect to receive your revised manuscript by Jul 14 2020 11:59PM. Please email us (plosmedicine@plos.org) if you have any questions or concerns.

***Please note while forming your response, if your article is accepted, you may have the opportunity to make the peer review history publicly available. The record will include editor decision letters (with reviews) and your responses to reviewer comments. If eligible, we will contact you to opt in or out.***

We ask every co-author listed on the manuscript to fill in a contributing author statement, making sure to declare all competing interests. If any of the co-authors have not filled in the statement, we will remind them to do so when the paper is revised. If all statements are not completed in a timely fashion this could hold up the re-review process. If new competing interests are declared later in the revision process, this may also hold up the submission. Should there be a problem getting one of your co-authors to fill in a statement we will be in contact. YOU MUST NOT ADD OR REMOVE AUTHORS UNLESS YOU HAVE ALERTED THE EDITOR HANDLING THE MANUSCRIPT TO THE CHANGE AND THEY SPECIFICALLY HAVE AGREED TO IT. You can see our competing interests policy here: http://journals.plos.org/plosmedicine/s/competing-interests.

Please use the following link to submit the revised manuscript:

https://www.editorialmanager.com/pmedicine/

Your article can be found in the "Submissions Needing Revision" folder.

To enhance the reproducibility of your results, we recommend that you deposit your laboratory protocols in protocols.io, where a protocol can be assigned its own identifier (DOI) such that it can be cited independently in the future. For instructions see http://journals.plos.org/plosmedicine/s/submission-guidelines#loc-methods.

Please ensure that the paper adheres to the PLOS Data Availability Policy (see http://journals.plos.org/plosmedicine/s/data-availability), which requires that all data underlying the study's findings be provided in a repository or as Supporting Information. For data residing with a third party, authors are required to provide instructions with contact information for obtaining the data. PLOS journals do not allow statements supported by "data not shown" or "unpublished results." For such statements, authors must provide supporting data or cite public sources that include it.

We look forward to receiving your revised manuscript.

Sincerely,

Emma Veitch, PhD

PLOS Medicine

On behalf of Clare Stone, PhD, Acting Chief Editor,

PLOS Medicine

plosmedicine.org

-----------------------------------------------------------

Requests from the editors:

*We'd suggest revising the title according to PLOS Medicine's style - after the study objective/question (in the first phrase of the title), we'd normally have a colon and then the study design, eg here "xxyy: retrospective cohort").

**Response from the authors:**

Thank you for your comments. We had changed the title to “Association between prehospital time and outcome of trauma patients in Asia: A cross-national, multicenter, retrospective cohort study “

**Changes made (Please refer to the marked version):**

Page 1, Line 2: A cross-national, multicenter, retrospective cohort study

*Please structure the abstract using the PLOS Medicine headings (Background, Methods and Findings, Conclusions) - "Methods and Findings" is a single subsection heading.

**Response from the authors:**

Thank you. We had changed the heading “Objective” to “Background.” “Method and Findings” was already used as a single subsection heading in the original version.

**Changes made (Please refer to the marked version):**

Page 4, Line 2: (Abstract) “Background”

*In the last sentence of the Abstract Methods and Findings section, please include a brief summary of any of the key limitation(s) of the study's methodology.

**Response from the authors:**

Thank you. We have added the key limitation in the abstract as below.

**Changes made (Please refer to the marked version):**

Page 4, Line 19: The key limitation of our study is the inherent bias, especially due to missing data as part of design of a retrospective cohort study.

*Currently in the abstract conclusions, the paper states "Long prehospital time ... but significantly affected the functional outcome of injured patients". However, this seems to go beyond what the retrospective cohort design is capable of concluding, ie this makes an assertion about causality rather than stating the findings as associations or links only. We'd suggest rephrasing this (and elsewhere in the paper) where a causal inference is made on the basis of observational data.

**Response from the authors:**

Thank you. It is indeed not appropriate to assertively state a causal inference based on our observational data. We have made revisions accordingly throughout the manuscript.

**Changes made (Please refer to the marked version):**

Page 4, Line 22: (Abstract) Longer prehospital time was not associated with an increased risk of 30-day mortality but it may increase the risk of poor functional outcomes in injured patients. We supported the concept of “golden hour” for trauma patients during prehospital care in Asia.

Page 14, Line 1: Multivariable logistic regression with overall patient data (n = 24,365) revealed that the RT was not associated with increased risk of 30-day mortality…

Page 16, Line 5: However, increased TPT, RT, or SH may be associated with increased risk of poor functional outcomes.

Page 17, Line 10: Our study showed that patients who experienced transportation delays were likely to have poorer functional outcomes.

Page 23, Line 3: our study suggested rapid transportation for all injured patients because longer prehospital time may be associated with an increased risk of poor functional outcomes

Page 23, Line 8: We supported the concept of the “golden hour” for trauma patients during prehospital care in Asia

*Ideally please reformat the in-text reference citations to use sequential numerals in square brackets (eg, [1], [2], etc).

**Response from the authors:**

Sorry for the error.

The reference style was changed to PLOS Medicine preferred style.

**Changes made (Please refer to the marked version):**

All citations and reference formats have been changed.

*Please clarify in the paper if the analysis reported here corresponds to one that was laid out in a prospectively-developed protocol or analysis plan? Please state this (either way) early in the Methods section.

**Response from the authors:**

Thanks for your comment.

Our study used the cross-national, multiple centers, and registry data of trauma patients from the Pan-Asia Outcome Study (PATOS) collaboration initiating in 2015 [1; already cited in the manuscript as [17]] and releasing phase 1 data in Dec 2019. The PATOS committee and principal investigators held conferences across Asian countries two to three times per year to discuss and approve the study proposals submitted by researchers from every PATOS site. The proposal of our study had been approved in the Taipei PATOS meeting, Nov 7, 2015.

Reference

[1]	Kong SY, Shin SD, Tanaka H, Kimura A, Song KJ, Shaun GE, et al. Pan-Asian Trauma Outcomes Study (PATOS): Rationale and Methodology of an International and Multicenter Trauma Registry. Prehospital Emergency Care. 2018;22(1):58-83.

**Changes made (Please refer to the marked version):**

Page 9, Line 5: (Method) The study proposal and analysis plan had been approved in PATOS Taipei meeting, Nov 7, 2015. The original study proposal is shown in supplement Table E1.

a) If a prospective analysis plan (from your funding proposal, IRB or other ethics committee submission, study protocol, or other planning document written before analyzing the data) was used in designing the study, please include the relevant prospectively written document with your revised manuscript as a Supporting Information file to be published alongside your study, and cite it in the Methods section. A legend for this file should be included at the end of your manuscript.

**Response from the authors:**

Thank you. We have added the original study proposal as a Supporting file, and get it cited in the Methods section.

**Changes made (Please refer to the marked version):**

Page 9, Line 5: (Methods) The study proposal and analysis plan had been approved in PATOS Taipei meeting, Nov 7, 2015. The original study proposal is shown in supplement Table E1.

b) If no such document exists, please make sure that the Methods section transparently describes when analyses were planned, and when/why any data-driven changes to analyses took place.

**Response from the authors:**

Thank you. We have added the document for originally designed analysis plan in the manuscript.

**Changes made (Please refer to the marked version):**

Page 9, Line 5: (Methods) The study proposal and analysis plan had been approved in PATOS Taipei meeting, Nov 7, 2015. The original study proposal is shown in supplement Table E1.

c) In either case, changes in the analysis-- including those made in response to peer review comments-- should be identified as such in the Methods section of the paper, with rationale.

**Response from the authors:**

Thank you for your advice.

The PATOS trauma database is characterized by an EMS-based registry. The initial proposal (2015) of current study planned to analyze the association between “the golden hour (i.e. injury time to definite care in hospital)” and trauma patient outcomes. However, when the PATOS phase 1 data was released in 2019, we found that there was an inadequate sample size of patients with ISS≥16.

Since the PATOS is an EMS-based trauma registry, we decided to revise the analysis plan focusing on the association between prehospital timelines and outcome and those factors associated with longer prehospital time.

Although it would be more specific to investigate the impact of prehospital time in major trauma patients with ISS≥16, we would lose power in the statistical analysis due to inadequate sample size, with a residual case number of 1,638 and 1,050 for the 30-day mortality and functional outcome status, respectively. The results of the subgroup analysis of major trauma patients with ISS≥16 had been presented in the original Figures 2 and 3, with the adjusted ORs of 1.00 (95%CI 0.93–1.08) on 30-day mortality and 0.99 (95%CI 0.94–1.04) on poor functional outcome.

Nevertheless, it is worth noting that the definition of major trauma is based on ISS scores, which is finally calculated at hospital discharge, not in the field by the emergency medical technicians (EMTs). Since our study was aimed at the prehospital time and prehospital care for patients with trauma, it is reasonable and actually more applicable if we take all trauma patients into consideration, because the results can simply remind the EMTs at the scene to scoop and run, and speed up the transportation of all trauma patients to avoid the risk of poor functional outcome.

Thanks again for your insightful suggestion. We have added it into the Methods Section and Discussion Section.

**Changes made (Please refer to the marked version):**

Page 9, Line 5: (Methods) The study proposal and analysis plan had been approved in PATOS Taipei meeting, Nov 7, 2015. The original study proposal is shown in supplement Table E1. The PATOS trauma database is characterized by an EMS-based registry. The initial proposal (2015) of current study planned to analyze the association between “the golden hour (i.e. injury time to definite care in hospital)” and trauma patient outcomes. However, when the PATOS phase 1 data was released in 2019, we found that there was an inadequate sample size of patients with ISS≥16, therefore the analysis plan was revised to test the association between prehospital timeliness and outcome.

Page 19, Line 9: (Discussion) We truly appreciate peer reviewers in putting forward some insightful concerns of our study. One of them considered it reasonable to more specifically investigate patients with major trauma (ISS ≥ 16) because they are more likely to be affected by prehospital time factors. Although it will be more specific to investigate the impact of prehospital time in major trauma patients with ISS>16, we will lose power in the statistical analysis due to inadequate sample size. The results of the subgroup analysis of major trauma patients with ISS>16 did not show association between prehospital time and outcome. It is also worth noting that the definition of major trauma is based on the ISS scores, which is finally calculated at hospital discharge, and not in the field by the emergency medical technicians (EMTs). Since our study aimed at the prehospital time and prehospital care for patients with trauma, it is reasonable and actually more applicable if we take all trauma patients into consideration, because the results can simply remind the EMTs at the scene to scoop and run, and speed up the transportation of all trauma patients to avoid the risk of poor functional outcome.

*In the Methods section where the authors say multivariate, do they mean multivariable?

**Response from the authors:**

Yes, we do mean multivariable and has made changes in the whole manuscript. Thank you for reminding us.

**Changes made (Please refer to the marked version):**

Page 11, Line 19: Multivariable logistic regression was used…

Page 11, Line 21: Variables that had a p <0.10 on the chi-square and Mann-Whitney U tests were selected for multivariable logistic regression analysis…

Page 12, Line 3: …the Mann-Whitney U test were selected for a multivariable linear regression analysis…

Page 14, Line 1: Multivariable logistic regression with overall patient data…

Page 14, Line 24: Multivariable logistic regression showed ….

Page 16, Line 17: … our study included as many confounders in the multivariable logistic regression as possible.

Figure 2 & Legend: Co-variate used in the multivariable logistic regression…

Figure 3 & Legend: Co-variate used in the multivariable logistic regression…

Table 2: TPT was put in the multivariable logistic regression …

Table 3: TPT was put in the multivariable logistic regression…

*In the Discussion section, more care is needed in a number of places with regard to causal inference - as noted above. For example, "However, increased TPT, RT, or SH may significantly increase the risk of poor functional outcomes" - this would be better stated as "associated with increased risk of...". Similarly, “our study suggested rapid transportation for all injured patients because increased prehospital time may lead to poor functional outcomes" could be rephrased more precisely.

**Response from the authors:**

Thank you for your advice.

We have accordingly made the changes in our manuscript.

**Changes made (Please refer to the marked version):**

Page 4, Line 22: (Abstract) Longer prehospital time was not associated with an increased risk of 30-day mortality but it may increase the risk of poor functional outcomes in injured patients. We supported the concept of “golden hour” for trauma patients during prehospital care in Asia.

Page 14, Line 1: Multivariable logistic regression with overall patient data (n = 24,365) revealed that the RT was not associated with increased risk of 30-day mortality…

Page 16, Line 5: However, increased TPT, RT, or SH may be associated with increased risk of poor functional outcomes.

Page 17, Line 10: Our study showed that patients who experienced transportation delays were likely to have poorer functional outcomes.

Page 23, Line 3: our study suggested rapid transportation for all injured patients because longer prehospital time may be associated with an increased risk of poor functional outcomes

Page 23, Line 8: We supported the concept of the “golden hour” for trauma patients during prehospital care in Asia.

-----------------------------------------------------------

Comments from the reviewers:

Reviewer #1: Thank you very much for the opportunity to review a manuscript titled 'Association between prehospital time and outcome of trauma patients in Asia: Does the "golden hour" matter?' This study investigated the association between prehospital time factors and survival and functional outcomes using data from several countries in Asia. The authors found an increasing risk for adverse functional outcome as prehospital time increases. The methods used in the study appear to be appropriate and the results seem to be reasonable. However, I have a concern re characteristics of patients included. 93.3% of the patients had ISS<16, which is deemed as non-major trauma. Therefore, outcomes of most participants are less likely to be affected by prehospital time factors. I feel that prehospital time factors may be more important for major trauma patients. It would be useful if you could repeat this analysis by including only major trauma patients, but I understand sample size will significantly decrease by doing so and you will lose power.

**Response from the authors:**

Thank you for the comments.

Although it would be more specific to investigate the impact of prehospital time in major trauma patients with ISS≥16, we would lose power in the statistical analysis due to inadequate sample size, with a residual case number of 1,638 and 1,050 for the 30-day mortality and functional outcome status, respectively. The results of the subgroup analysis of major trauma patients with ISS≥16 had been presented in the original Figures 2 and 3, with the adjusted ORs of 1.00 (95%CI 0.93–1.08) on 30-day mortality and 0.99 (95%CI 0.94–1.04) on poor functional outcome.

Nevertheless, it is worth noting that the definition of major trauma is based on ISS scores, which is finally calculated at hospital discharge, not in the field by the emergency medical technicians (EMTs). Since our study was aimed at the prehospital time and prehospital care for patients with trauma, it is reasonable and actually more applicable if we take all trauma patients into consideration, because the results can simply remind the EMTs at the scene to scoop and run, and speed up the transportation of all trauma patients to avoid the risk of poor functional outcome.

Thanks again for your insightful suggestion.

We have added it into the Methods Section and Discussion Section.

**Changes made (Please refer to the marked version):**

Page 19, Line 9: We truly appreciate peer reviewers in putting forward some insightful concerns of our study. One of them considered it more reasonable to more specifically investigate patients with major trauma (ISS ≥ 16) because they are more likely to be affected by prehospital time factors. Although it will be more specific to investigate the impact of prehospital time in major trauma patients with ISS≥16, we will lose power in the statistical analysis due to inadequate sample size. The results of the subgroup analysis of major trauma patients with ISS>16 did not show association between prehospital time and outcome. It is also worth noted that the definition of major trauma is based on the ISS scores, which is finally calculated at hospital discharge, not in the field by the emergency medical technicians (EMTs). Since our study aimed at the prehospital time and prehospital care for patients with trauma, it is reasonable and actually more applicable if we take all trauma patient into consideration, because the results can simply remind the EMTs at the scene to scoop and run, and speed up the transportation of all trauma patients to avoid the risk of poor functional outcome.

My other minor concerns are described below.

The 2nd last sentence in a paragraph with a header of 'Study design and setting', Malaysia appears twice in the sentence.

**Response from the authors:**

Thanks for your attentiveness. We have deleted the redundant one.

**Changes made (Please refer to the marked version):**

Page 8, Line 12: This cross-national trauma registry consisted of 33 sites in 14 Asian countries such as China, India, Korea, Japan, Malaysia, Thailand, the Philippines, Taiwan, and Vietnam.

Do all participating countries use the same version of the AIS to compute ISS? If not, please address the issue.

**Response from the authors:**

Thank you for the advice.

Among the participating countries considered in final analysis, Korea, Japan, and Malaysia use the AIS 2005 version update 2008 (7-digit codes) while Taiwan uses the AIS 1995 version (5-digits) to compute ISS.

**Changes made (Please refer to the marked version):**

Page 9, Line 25: (Methods) Among the participating countries considered in final analysis, Korea, Japan, and Malaysia use the AIS 2005 version update 2008 (7-digit codes) while Taiwan uses the AIS 1995 version (5-digits) to compute ISS.

In 'Statistical analysis': 'Continuous **dependent** variables were compared using non-parametric ANOVA and Mann-Whitney U test. Categorical and nominal **dependent** variables were compared using the chi-square and Pearson Chi-square test or Fisher's exact test.'

I may be misunderstanding, but your dependent variables are 'mortality' and 'functional outcome', aren't they? I presume you meant to say 'independent variable'? Apology if I misunderstood.

**Response from the authors:**

Thank you. We have deleted the misunderstanding words per your advice.

**Changes made (Please refer to the marked version):**

Page 11, Line 15: Continuous variables were compared using non-parametric ANOVA or Mann-Whitney U test. Categorical and nominal variables were compared using Pearson Chi-square test or Fisher’s exact test.

'Continuous dependent variables were compared using non-parametric ANOVA **and** Mann-Whitney U test.' I think you tested using either methods. So, it should be 'ANOVA **or** Mann-Whitney'

**Response from the authors:**

Yes, it was a grammar error. Thank you very much!

**Changes made (Please refer to the marked version):**

Page 11, Line 15: Continuous variables were compared using non-parametric ANOVA or Mann-Whitney U test. Categorical and nominal variables were compared using Pearson Chi-square test or Fisher’s exact test.

'Categorical and nominal dependent variables were compared using the chi-square and Pearson Chi-square test or Fisher's exact test.' -> What is the difference between 'chi-square test' and 'Pearson Chi-square test'? Did you use other type of chi-square test than Pearson chi-square?

**Response from the authors:**

Thank you. We have deleted the surplus word “chi-square”.

**Changes made (Please refer to the marked version):**

Page 11, Line 15: Continuous variables were compared using non-parametric ANOVA or Mann-Whitney U test. Categorical and nominal variables were compared using Pearson Chi-square test or Fisher’s exact test.

'To determine the potential factors that may influence scene to hospital time, variables that had a p <0.10 on the Mann-Whitney U test were selected for a multivariate linear regression analysis using the forced entry method.'

It is not clear why you derived a linear regression model because the 2 outcome variables are dichotomous (mortality and functional outcome).

**Response from the authors:**

Thank you for your comments.

The linear regression model was used in the post-hoc analysis of potential factors associated with total prehospital time, which is a continuous dependent variable.

We apologize that we did not mention the post-hoc analysis in the hypothesis and Method section. We have amended it in the current edition. We also found some typo errors taking “total prehospital time” as “scene to hospital time” in the manuscript and have corrected it.

Thank you for the important advice.

**Changes made (Please refer to the marked version):**

Page 7, Line 26: Additionally, we tried to investigate potential factors which may influence prehospital time. Considering that longer prehospital time is detrimental to patients’ outcome, the findings of this study could further guide the EMS in shortening prehospital time.

Page 12, Line 1: To determine the potential factors that may influence total prehospital time, variables that had a p <0.10 on the Mann–Whitney U test were selected for a multivariable linear regression analysis, as the dependent variable is continuous, using the forced entry method.

Please explain why 'scene to hospital time', not total prehospital time or scene time, was used as a dependent variable in the linear regression.

**Response from the authors:**

Thank you for reminding us the mistake.

The dependent variable used in the linear regression in our study was actually total prehospital time and was miswritten in some sentences. We have corrected them.

**Changes made (Please refer to the marked version):**

Page 12, Line 1: To determine the potential factors that may influence total prehospital time, variables that had a p <0.10 on the Mann–Whitney U test were selected for multivariable linear regression analysis, as the dependent variable is continuous, using the forced entry method.

Page 15, Line 20: Potential factors influencing total prehospital time

'The restricted cubic spline regression of prehospital time and predicted possibilities of favourable functional outcome are depicted in Figure 4.' in Functional outcome at discharge in the results-> This is not described in the Methods.

**Response from the authors:**

Sorry that we did not describe it in the Method section

We have corrected it in the manuscript.

**Changes made (Please refer to the marked version):**

Page 11, Line 25: The association of prehospital time and predicted possibilities of favorable functional outcome was presented using the restricted cubic spline regression.

The authors reported factors associated with total prehospital time, but this is not part of aims. It is ok to analyse the association, but the authors need to present it as an aim and give some rationale to investigate it if possible. Otherwise, I was unsure why the authors derived the linear regression model.

**Response from the authors:**

As mentioned above, we apologize that we did not mention the post-hoc analysis in the hypothesis and Method section. Our primary goal was to study the association between prehospital time and outcome of injured patients, and post-hoc analysis aimed at the factors affecting the prehospital time so that we could guide EMTs to shorten the prehospital time in the field.

We have amended it in our manuscript. And because determining factors to prehospital time was not the primary goal, we just mentioned it briefly in the Result section and put the relevant table as the supplemental file.

**Changes made (Please refer to the marked version):**

Page 7, Line 26: Additionally, we tried to investigate potential factors which may influence prehospital time. Considering that longer prehospital time is detrimental to patients’ outcome, the findings of this study could further guide the EMS in shortening prehospital time.

I could not find Table E1, E2, E4. There is no Table E3.

**Response from the authors:**

Thank you. Tables E1~E6 were in the supplementary file. Sorry for that table E3 was miswritten as “eTable 3” in previously submitted manuscript. And the previous table E3 was now Table E4 after revision.

We have clarified it.

**Changes made (Please refer to the marked version):**

Page 15, Line 26: Table E4

-----------------------------------------------------------

Reviewer #2: The authors have conducted a retrospective cohort study of trauma patients transported to hospitals by emergency medical service (EMS) between 2016 and 2018 using data from the Pan-Asia Trauma Outcomes study (PATOS) database.

Comments:

Is the timeframe of the data (2016-2018) still relevant in today's clinical setting? Has medical equipment available in transit changed since then, for instance?

The authors acknowledge that "... there have been significant advances in resuscitation, airway management, circulatory access, and hemorrhage control as the prehospital care provided by paramedics and other emergency medical technicians".

**Response from the authors:**

Thank you for your comments.

Our study is based on the PATOS registry data from 2016 to 2018. Although there have been significant advances in resuscitation interventions in the prehospital setting, these might be observed in some major cities, however, it is not the case in prehospital care as part of daily EMS practices across Asia. For example, until now less than half of Asian EMS cities have approved the prehospital intubation by paramedics, not to mention other advanced care like the hemostasis medication tranexamic acid (TXA) or prehospital transfusion of blood components. Instead, advanced interventions for trauma patients in the prehospital setting will theoretically prolong the prehospital time and are likely to impair the patient outcome according to our findings.Therefore, we think the study results based on the PATOS registry data from 2016 to 2018 still make sense under these circumstances.

We have added your concern in the Discussion Section.

**Changes made (Please refer to the marked version):**

Page 18, Line 5: (Discussion) Our study is based on the PATOS registry data from 2016 to 2018. Although there have been significant advances in resuscitation interventions in the prehospital setting, these might be observed in some major cities, however, it is not the case in prehospital care as part of daily EMS practices across Asia. Instead, advanced interventions for trauma patients in the prehospital setting will theoretically prolong the prehospital time and are likely to impair the patient outcome according to our findings.

Whilst briefly mentioned in the limitations, how do the authors expect excluding patients based on missing values might have affected their results?

Can these patients/values be assumed to be missing at random? Are the remaining patients included in the analysed sample representative of the wider population?

**Response from the authors:**

A total of 48,348 patients were transported from the scene to hospital by EMS from 2016 to 2018. Unreasonable data and data without necessary variables were noted as missing in the Method section, resulting in 23,983 patients excluded due to missing data.

As per your advice, we further compared the basic characteristics of included sample and excluded sample in Table E6. Unfortunately, patients’ age, severity, response time, scene to hospital time, and outcome really differed between two groups. This selection bias should be one of the inherent limitations of the study’s retrospective design. Also, the main missing values were the prehospital time intervals which we aimed to investigate in our study, so it will be even more unreasonable to perform imputation of those missing data. However, the main difference between the two samples were the outcome variables. As the emergency medical technicians (EMTs) who recorded the prehospital time variables may not be able to predict the patients’ outcome, much of this missing data might be reasonably seen as a random missing, for which it cannot be intentionally manipulated.

Eventually, although we excluded half of the samples in our analysis, we still had investigated more than 20 thousand patients in this cross-national, multi-center cohort study, which should still be valuable and informative contribution to the knowledge gap in this area.

**Changes made (Please refer to the marked version):**

Page 21, Line 11: After enrolling those patients with all valid data and excluding unreasonable data and outliers, the final dataset may have been affected by selection bias. The main missing values were the prehospital time intervals which we aimed to investigate in our study, so it would be even more unreasonable to perform imputation of those missing data. We further compared the basic characteristics of the included and excluded sample due to missing data and found differences in patients’ age, severity, response time, scene to hospital time, and outcome (Table E6). However, the main difference between the two samples were the outcome variables. As the emergency medical technicians (EMTs) who recorded the prehospital time variables may not be able to predict the patients’ outcome, much of this missing data might be reasonably seen as a random missing, for which it cannot be intentionally manipulated. Eventually, although we excluded half of the samples in our analysis, we still had investigated more than 20 thousand patients in this cross-national, multi-center cohort study, which should still be valuable and informative contribution to the knowledge gap in this area.

"...age was below 1 year or above 120 years, which were considered unreasonable data and potential outliers."

Can the authors explain why age below 1 was considered an outlier?

**Response from the authors:**

Sorry for the typo error. We have corrected it.

The statistic plan was to exclude those data with below 0 years old, which was certainly unreasonable.

**Changes made (Please refer to the marked version):**

Page 10, Line 19: age was below 0 year or above 120 years

Did the authors consider variability in the time measurements they included, and how uncertainty in measurements might impact their results?

**Response from the authors:**

The advantage of our study is the cross-national, multi-center, large-scale cohort with retrospective registry, as well as its limitation inherent in the study design.

The tools for time measurement indeed varied from site to site. Data from Taiwan, Japan, and Korea used a centralized time on ambulance run sheets and electric medical records of all time variables to ensure the accuracy. However, some PATOS sites in Malaysia were not equipped with a centralized timer in their measurement and in records of ambulance run sheets, thus the random errors could not be totally excluded. This should be one of the limitations in our study.

Theoretically the variability in the time measurements might not be intentional, hence it is not unidirectional to the hypothesis. We think it is a kind of random error and could be reasonably minimized by a large sample size.

In the subgroup analysis (Figure 2 & 3), we investigated the association of prehospital time and outcome in different countries. As shown in the figure, longer total prehospital time was not associated with an increased risk of 30-day mortality and consistently presented the same trend of increasing risk of poor functional outcome in each country despite the potential difference in time measurements. This should be another evidence to support our viewpoint.

Thanks again for your insightful comments.

We have discussed this in the Discussion section.

**Changes made (Please refer to the marked version):**

Page 19, Line 22: Another concern risen in the peer review was aimed at the potential bias caused by variability in the time measurements of different countries. Truly, the tools for time measurement indeed varied from site to site. In countries such as Taiwan, Japan and Korea use of centralized timer on their electric medical records and ambulance run sheets were used to ensure the accuracy. However, some PATOS sites in Malaysia were not equipped with a centralized timer in their measurement and records of ambulance run sheets, thus random errors could not be totally excluded. We think this kind of random error could be reasonably minimized by using a large sample size. Besides, subgroup analysis has shown that longer total prehospital time was not associated with increased risk of 30-day mortality and presented the same trend of increasing risk of poor functional outcome in each country despite the potential difference in time measurements.

The statistical techniques and tests used appear to be appropriate for the data type and research question.

**Response from the authors:**

Thank you very much.

We truly appreciate your compliments.

How sensitive are the findings of the logistic regression to the cut off of 30-day mortality? Do 20- or 40- day mortality models show any substantial difference in the results?

Albeit the authors claim that "30-day survival status is considered the standard follow-up outcome for trauma patients."

**Response from the authors:**

We set the “30-day survival status” as the primary outcome in our study because the literature suggested it as the standard follow-up outcome for trauma patients according to the Utstein template. [1; cited in the manuscript as [25]]

As per your suggestions, we have performed a sensitivity analysis of mortality within 24 hours, 14 days, 30 days, or 60 days. The result also supported that longer prehospital time may not be associated with increased risk of mortality. Only longer SH was shown to increase the risk of 60-day mortality, but longer follow up period may be subjected to more potential bias and inadequate statistic power to detect the true difference of hypothesis. (As presented in the appendix table below)

Because the 30-day mortality was set as the primary outcome measurement in our study, detailed data of the sensitivity analysis was not presented in our article to avoid the problem of multiple comparison.

Reference

[1] Ringdal KG, Coats TJ, Lefering R, Di Bartolomeo S, Steen PA, Roise O, et al. The Utstein template for uniform reporting of data following major trauma: a joint revision by SCANTEM, TARN, DGU-TR and RITG. Scand J Trauma Resusc Emerg Med. 2008;16:7.

| **Appendix.** Sensitivity analysis of the association of prehospital time on mortality within different time period, adjusted odds ratio (95% confidence interval) | | | | |
| --- | --- | --- | --- | --- |
|  | 24 hours | 14 days | 30 days | 60 days |
| n (%) | 92 (0.4) | 200 (0.8) | 280 (1.1) | 287 (1.2) |
| RT (per 10 min) | 0.87 (0.70-1.07) | 0.93 (0.76-1.14) | 0.99 (0.92-1.06) | 0.98 (0.92-1.06) |
| SH (per 10 min) | 0.87 (0.72-1.04) | 0.91 (0.78-1.07) | 1.08 (1.00-1.17) | 1.10 (1.01-1.20) |
| TPT (per 10 min) | 0.89 (0.78-1.01) | 0.92 (0.81-1.04) | 1.03 (0.98-1.09) | 1.04 (0.98-1.10) |
| RT=response time, SH=scene to hospital time, TPT=total prehospital time | | | | |

**Changes made (Please refer to the marked version):**

Page 20, Line 15: Several reviewers had mentioned the sensitivity of the findings of the logistic regression to short term mortality (24-hour) or mortality within different time period. In response to reviewers’ suggestions, we have performed a sensitivity analysis on different outcomes: mortality within 24 hours, 14 days, 30 days, or 60 days. The results consistently supported that longer prehospital time may not be associated with increased risk of mortality.

Did the authors explore and include interaction variables within their regression modelling?

**Response from the authors:**

Thank you for your opinion.

We did not include the interaction variables in our regression model. The reason is that there was no significant difference in the result of subgroup analysis between different subgroups (Figure 2 & 3). Therefore, interactions between different variables were expected to be low and may not change the current result.

Did the authors consider differences by the four included countries, or just look at the pooled Asia scenario?

**Response from the authors:**

Thank you for your suggestion.

We had performed subgroup analysis of each country as represented in Figure 2 & 3.

As shown in the figure, longer total prehospital time was not associated with increased risk of 30-day mortality and consistently presented the same trend of increasing risk of poor functional outcome in each country. The difference between each included country seemed to be low as compared to the pooled Asia scenario.

We will make a revision in Figure 2 & 3 to make it more clarified.

**Changes made (Please refer to the marked version):**

Please refer to revised Figure 2 & 3.

Page 20, Line 24: Finally, the potential differences between different countries and hospitals was also of concern to several reviewers, and one of them suggested the use of hierarchical nested models that account for patient clustering. However, we performed many subgroup analyses, and found that longer total prehospital time was not associated with increased risk of 30-day mortality and consistently presented the same trend in increasing risk of poor functional outcome in each country. The difference between each included country seemed to be low as compared to the pooled data for Asia. Furthermore, hospitals enrolled in the PATOS registry were all teaching hospitals capable of trauma resuscitation. In light of the above reasons, we assumed the difference among different countries and hospitals may be small.

-----------------------------------------------------------

Reviewer #3: The research question and topic are certainly of interest. The authors present a multi-county evaluation of Asian trauma patients and the association of prehospital time with mortality and functional status at discharge.

The background is logical but too long. The authors review several individual prior studies on prehospital time in detail that distracts from focusing on their objectives and hypothesis. This review of the existing literature would be better served in the discussion of the paper.

**Response from the authors:**

Thank you. We will prune the background section according to your suggestions. Please refer to the Background section of the revised manuscript

**Changes made (Please refer to the marked version):**

Page 6, Line 19: Most of them showed no significant correlation or even an inverse correlation. [7, 8]

Page 6, Line 25: several articles aimed to identify specific injury subgroups that may benefit from reduced prehospital time such as younger patients or patients with TBI. [9, 10]

The authors clearly outline the objectives. The authors should explicitly state their hypothesis regarding the relationship between prehospital time and outcomes in their population.

**Response from the authors:**

Thank you. We will include our hypothesis at the end of the background section.

**Changes made (Please refer to the marked version):**

Page 7, Line 24: We hypothesized that trauma patients can be benefited by rapid transportation, and the concept of golden hour could still be applicable in prehospital care for trauma in Asia. Additionally, we tried to investigate potential factors which may influence prehospital time.

The methods require some clarification and I have several concerns as below:

1. The database used for the study requires further description. Which hospitals submit data to the database? All hospitals, only trauma centers? Is data submission voluntary or required of trauma centers? Do the types of hospitals include vary across countries in the database?

**Response from the authors:**

Thank you for your comments.

The rationale, methodology, and background of PATOS database has been described in previously published PATOS paper [1, 2]. Among the final data included in this study from Korea, Japan, Taiwan and Malaysia, all hospitals participating in the investigation are academic teaching hospitals with the capability of trauma resuscitation. The participation of PATOS registry is voluntary. However, the category of hospital accreditation is different from countries to countries. For example, there was no designated separated level 1 trauma center in Korea and Malaysia. Among PATOS sites in Korea, as of July 2020, there are four trauma centers, three regional emergency medical centers, and five local emergency medical centers among the 12 hospitals under investigation. Hospitals in Malaysia do not have trauma center designation too. The hospitals involved in PATOS are able to manage most of the trauma except some that are without neurosurgical intervention. Data from Taiwan and Japan were all from voluntary trauma centers (tertiary hospitals).

We have added this to the manuscript.

References

[1] Kong SY, Shin SD, Tanaka H, Kimura A, Song KJ, Shaun GE, et al. Pan-Asian Trauma Outcomes Study (PATOS): Rationale and Methodology of an International and Multicenter Trauma Registry. Prehospital Emergency Care. 2018;22(1):58-83.

[2] Sun KM, Song KJ, Shin SD, Tanaka H, Shaun GE, Chiang WC, et al. Comparison of E mergency Medical Services and Trauma Care Systems Among Pan-Asian Countries: An International, Multicenter, Population-Based Survey. Prehospital emergency care. 2017;21(2):242-51.

**Changes made (Please refer to the marked version):**

Page 8, Line 15: (Methods) The participation of PATOS registry is voluntary.

Page 12, Line 26: (Results) All hospitals from these countries participating in the investigation are academic teaching hospitals with functional capability for trauma resuscitation.

2. Given variation in prehospital systems, the authors should provide a brief description of the EMS systems and capabilities in the studied countries. Are there significant differences between the counties in the EMS systems for the patients studied?

**Response from the authors:**

Thank you for your comments.

Most of the EMS and trauma care systems involved in this registry were in the urban cities of each country. Patients’ data would be recorded in the registry if they were sent to the participating hospitals due to trauma, either from the scene or via inter-hospital transport. The only exception was the data from Taiwan where the registry data had EMS-transported trauma patients that met the criteria of prehospital activation of major trauma.

We have explained this in the manuscript.

**Changes made (Please refer to the marked version):**

Page 8, Line 15: (Method - Study design and setting) The participation of PATOS registry is voluntary. Patients data would be recorded in the registry if they were sent to the participating hospitals due to trauma, either from scene or via inter-hospital transport. The only exception was data from Taiwan where the registry data had information of EMS transported trauma patients who met the criteria of prehospital activation for major trauma.

3. It is not clear from the methods if the patients included were only patients transported from the scene, patients transferred between hospitals, or both?

**Response from the authors:**

Thanks for your suggestions.

In the first sentence of the subsection of “Selection of participants,” we stated that only patients transported to the hospital by EMS will be included in our study.

We will clarify make this point better in the Abstract and Method section.

**Changes made (Please refer to the marked version):**

Page 4, Line 5: (Abstract) We conducted a retrospective cohort study of trauma patients transported from the scene to hospitals by emergency medical service (EMS) between 2016 and 2018 using data from the Pan-Asia Trauma Outcomes study (PATOS) database.

Page 8, Line 23: (Method) Patients were included if they were transported from the scene to hospital by the EMS during the period from 2016 to 2018.

4. The authors exclude nearly half of the eligible patients for missing data. The authors should consider multiple imputation which has been validated in similar large trauma registry databases to avoid systematic bias that may result from excluding all these patients. The authors should also compare patients included and excluded to evaluate for potential bias from excluding these patients

**Response from the authors:**

Thank you for the insightful advice.

The advantage of our study is the cross-national, multi-center, large-scale cohort with retrospective registry, as well as its limitation inherent in the study design. A total of 48,348 patients were transported from the scene to hospital by EMS from 2016 to 2018. Unreasonable data and data without necessary variables were noted as missing in the Method section, resulting in 23,983 patients excluded due to missing data.

As per your advice, we further compared the basic characteristics of included sample and excluded sample in Table E6. Unfortunately, patients’ age, severity, response time, scene to hospital time, and outcome really differed between two groups. This selection bias should be one of the major limitation of the study’s retrospective design. Also, the main missing values were the prehospital time intervals which we aimed to investigate in our study, so it will be even more unreasonable to perform imputation of those missing data. However, the main difference between the two samples were the outcome variables. As the emergency medical technicians (EMTs) who recorded the prehospital time variables may not be able to predict the patients’ outcome, much of this missing data might be reasonably seen as a random missing, for which it cannot be intentionally manipulated.

Eventually, although we excluded half of the samples in our analysis, we still had investigated more than 20 thousand patients in this cross-national, multi-center cohort study, which should still be valuable and informative contribution to the knowledge gap in this area.

**Changes made (Please refer to the marked version):**

Page 21, Line 11: After enrolling those patients with all valid data and excluding unreasonable data and outliers, the final dataset may have been affected by selection bias. The main missing values were the prehospital time intervals which we aimed to investigate in our study, so it would be even more unreasonable to perform imputation of those missing data. We further compared the basic characteristics of the included and excluded sample due to missing data and found differences in patients’ age, severity, response time, scene to hospital time, and outcome (Table E6). However, the main difference between the two samples were the outcome variables. As the emergency medical technicians (EMTs) who recorded the prehospital time variables may not be able to predict the patients’ outcome, much of this missing data might be reasonably seen as a random missing, for which it cannot be intentionally manipulated. Eventually, although we excluded half of the samples in our analysis, we still had investigated more than 20 thousand patients in this cross-national, multi-center cohort study, which should still be valuable and informative contribution to the knowledge gap in this area.

5. Why did the authors no evaluate transport time as one of the prehospital intervals (time from leaving the scene to arriving at the hospital)? This may be an important interval to evaluate as well independent of the total prehospital time.

**Response from the authors:**

Thank you for your precise viewpoints.

This study aimed to evaluate the association between prehospital time and the outcomes of injured patients. Among the prehospital time intervals in the PATOS database, one of the major countries (Korea) mostly reported the scene to hospital time instead of transport time in their uploaded data. We cannot exactly estimate the transport time in this part of the Korean data and excluding all of this would harm the statistic power to detect the hypothesis. Therefore, we chose “scene to hospital time” as one of the independent variables, not the isolated transport time.

We have clarified it by adding the forehand reason in the “Measurements-Variables” section.

**Changes made (Please refer to the marked version):**

Page 10, Line 6: (Method) The prehospital time intervals were extracted using prehospital timing with maximal valid value (Table E2). For the prehospital time intervals in the PATOS database, one of the major countries (Korea) mostly reported the scene to hospital time instead of transport time in their uploaded data. We could not exactly estimate the transport time using this part of the Korean data, also excluding all of this information would harm the statistic power required to detect the hypothesis. Hence, the time interval used in our study was response time ([RT] from the time of injury to the time of EMS arrival at scene), scene to hospital time ([SH] from the time of EMS arrival at the scene to the time of EMS arrival at the ED), and total prehospital time ([TPT] from the time of injury to the time of EMS arrival at ED).

6. The authors state a strength of the study was using time of injury rather than time of EMS activation. How this data was known and collected, and how reliable is it?

**Response from the authors:**

Thank you for the insightful comments.

The injury time is the core variable in the PATOS registry. In Korea, the time of injury is based on the emergency department physician (usually intern or resident) asking the patient and documenting it on the medical record. All participating hospitals in Korea use the Electronic Medical Record (EMR) system. When the patient was injured (or when symptoms occurred), it is an essential medical record evaluation item in Korea, which can be problematic if not correctly placed. PATOS has a quality control coordinator for each hospital. The time of injury is the core variable, and the official time entered in the EMR is used as it is.

In Malaysia, the time of injury was recorded when the caller or the patient is able to give the information. Otherwise, it will be an estimation of the EMT at the scene. For data from Japan and Taiwan, the injury time is documented on the ambulance run sheet then entered in the PATOS registry. We believe that the time of injury entered into the PATOS database is reliable under most circumstances, but the occurrence of random errors cannot be totally excluded.

We have added your concern in the manuscripts.

**Changes made (Please refer to the marked version):**

Page 16, Line 23: The injury time is the core variable in the PATOS registry. The data was usually obtained from caller or patient information. Otherwise, it would be an estimation of the EMT at the scene.

7. Did the authors consider a shorter-term mortality outcome such as 24 hours? Many factors between prehospital transport and 30 days may occur that obscure the impact of prehospital times on mortality

**Response from the authors:**

Thank you for giving us chance to clarify this.

Since our study aimed at the prehospital time and prehospital care for all patients with trauma, not only for those with major trauma (i.e. ISS > 16 at hospital discharge), a shorter-term mortality outcome such as 24 hours scarcely occurred in the finally analyzed population. In fact, only 92 (0.4%) cases passed away within 24 hours out of 24,365 enrollees. We think the case number is with inadequate power to test the hypothesis.

**Changes made (Please refer to the marked version):**

Page 20, Line 15: Several reviewers had mentioned the sensitivity of the findings of the logistic regression to short term mortality (24-hour) or mortality within different time period. In response to reviewers’ suggestions, we have performed a sensitivity analysis on different outcomes: mortality within 24 hours, 14 days, 30 days, or 60 days. The results consistently supported that longer prehospital time may not be associated with increased risk of mortality. Also, a shorter-term mortality outcome such as 24 hours scarcely occurred in the finally analyzed population. In fact, only 92 (0.4%) cases passed away within 24 hours out of 24,365 enrollees. We think the case number is with inadequate power to test the hypothesis.

8. Related to the above point, the authors do not adjust for any in-hospital variables that may confound mortality (e.g. complications, procedures, blood and crystalloid resuscitation volumes). The authors should adjust for these factors if evaluating 30-day mortality

**Response from the authors:**

Thanks for your suggestion. However, many in-hospital variables were not able to be included in the regression model either due to missing data or the variable was not recorded in the registry. This is one of the limitations of our study and has been listed in the limitation section.

On the other hand, although in-hospital variables will affect patients’ outcome, they are less likely to be potential confounders of the association between prehospital time and outcome because in-hospital treatment is theoretically dependent on patient’s condition, and not associated with the prehospital timeliness.

**Changes made (Please refer to the marked version):**

Page 22, Line 6: In-hospital variables such as complications, procedures, blood and crystalloid resuscitation volumes were not able to be included in our analysis either due to missing data or non-recording of the variable in the registry. We were unable to match all the confounders to determine the direct effect of prehospital time on outcome. On the other hand, although in-hospital variables will affect patients’ outcome, it is less likely that they are potential confounders of the association between prehospital time and outcome because in-hospital treatment is theoretically dependent on patient’s condition and is not associated with the prehospital timeliness.

9. Are patients that died before reaching the hospital or died at non-trauma centers or small community hospitals included in the database? I am concerned there is a survival bias in the dataset in which patients that had longer prehospital time may have died before reaching a hospital that includes data, and patients with long prehospital times in the dataset were at low risk of dying, making it look like there's no relationship or even that longer prehospital times were protective against death.

**Response from the authors:**

Thank you for your insightful comments.

Our study included only EMS patients with primary transportation (i.e. from the scene to hospitals), and those who collapsed in the ambulance transport were also recorded in the database because they would still be transported to the hospital.

Actually, trauma patients with out-of-hospital cardiac arrest (OHCA) only counted for a very small proportion in the finally analyzed cohort, with only 62 OHCA patients (less than 0.5%) having cardiac arrest before being sent to the hospital.

We believe this viewport is important, and have added it into one paragraph of the Discussion Section.

**Changes made (Please refer to the marked version):**

Page 20, Line 7: One of the reviewers mentioned a survival bias in the dataset in which patients that had longer prehospital time may have died before reaching a hospital and that this information could not be included in the data. Actually, patients who collapsed in the ambulance transport were also recorded in the database because they would still be transported to the hospital. Also, trauma patients with out-of-hospital cardiac arrest (OHCA) only counted for a very small proportion in the finally analyzed cohort, with only 62 OHCA patients (less than 0.5%) having cardiac arrest before being sent to the hospital, hence, the population of patients who died out of hospital and were not sent to the hospital should be scarce.

10. Do the authors know who administered the MRS and whether a structured questioner was used? Studies show poor interrater reliability without a structured questioner. This may be even more important since it has not robustly been validated in the general trauma population (only case-control study cited by the authors).

**Response from the authors:**

Thank you for the important viewpoint.

The MRS data from Korea and Japan was obtained by the physician (usually a resident or intern) with a structured questionnaire, while in Malaysia, data was collected by the nursing staff during discharge without a structured questionnaire. Data from Taiwan did not evaluate MRS during the period 2016 to 2018.

We have clarified this point in the manuscripts.

**Changes made (Please refer to the marked version):**

Page 10, Line 26: (Method) The MRS data from Korea and Japan was obtained by the physician (usually a resident or intern) with a structured questionnaire, while in Malaysia, data was collected by the nursing staff during discharge without a structured questionnaire. Data from Taiwan did not evaluate MRS during the period 2016 to 2018.

11. The authors should use models that account for patient clustering in the different countries and hospitals in the dataset (e.g. hierarchical nested models)

**Response from the authors:**

Thank you. Using hierarchical nested models as you mentioned was truly a precise way to investigate the difference caused by different countries and hospitals. However, in our study, we had studied the association between prehospital time and outcome in many subgroup analyses (Figure 2 & 3). As shown in the figure, longer total prehospital time was not associated with an increased risk of 30-day mortality and consistently presented the same trend of increasing risk of poor functional outcome in each country. The difference between each included country seemed to be low as compared to the pooled Asia scenario. Furthermore, hospitals enrolled in the PATOS registry were all tertiary medical centers. In sight of the above reasons, we assumed little difference among different countries and hospitals.

Thanks again for your advice again.

**Changes made (Please refer to the marked version):**

Page 20, Line 24: Finally, potential differences between different countries and hospitals was also of concern to several reviewers, and one of them suggested the use of hierarchical nested models that account for patient clustering. However, we performed many subgroup analyses, and found that longer total prehospital time was not associated with increased risk of 30-day mortality and consistently presented the same trend in increasing risk of poor functional outcome in each country. The difference between each included country seemed to be low as compared to the pooled data for Asia. Furthermore, hospitals enrolled in the PATOS registry were all teaching hospitals capable of trauma resuscitation. In light of the above reasons, we assumed the difference among different countries and hospitals may be small.

12. The authors should present discrimination and calibration assessments of their outcome models

**Response from the authors:**

Thank you for the suggestions.

We have tested the discrimination and calibration using the ROC curve and Hosmer–Lemeshow test. The result has been reported in the revised manuscript.

Our study showed good discrimination but inadequate calibration. However, the literature also reported that all goodness of fit test may have decreased power in the study with large-scale sample size, which might explain the inadequate calibration in our regression model. [1, 2] (cited in the manuscript as [40, 41])

Reference

[1].	Kramer AA, Zimmerman JE. Assessing the calibration of mortality benchmarks in critical care: The Hosmer-Lemeshow test revisited. Critical care medicine. 2007;35(9):2052-6. Epub 2007/06/15.

[2].	Afessa B, Gajic O, Morales IJ, Keegan MT, Peters SG, Hubmayr RD. Association between ICU admission during morning rounds and mortality. Chest. 2009;136(6):1489-95.

**Changes made (Please refer to the marked version):**

Page 11, Line 23: The discrimination of the regression model was tested using the area under the receiver operating characteristic curve for each outcome.

Page 14, Line 11: The area under the receiver operating characteristic curve (AUROC) of the multiple logistic regression model for association of RT, SH and 30-day mortality was 0.951 (95% CI: 0.935–0.966) and the Hosmer–Lemeshow test showed inadequate fit (χ2 = 20.696, p = 0.008). The AUROC of the multiple logistic regression model for association of TPT and 30-day mortality was 0.951 (95% CI: 0.937–0.966) and the Hosmer–Lemeshow test showed inadequate fit (χ2 = 19.975, p = 0.01).

Page 15, Line 2: The AUROC of the multiple logistic regression model for association of RT, SH and functional outcome at discharge was 0.768 (95% CI: 0.755–0.781) and the Hosmer–Lemeshow test showed inadequate fit (χ2 = 37.886, p < 0.001). The AUROC of the multiple logistic regression model for association of TPT and functional outcome at discharge was 0.769 (95% CI: 0.755–0.782) and the Hosmer–Lemeshow test showed inadequate fit (χ2 = 36.552, p < 0.001).

Page 22, Line 19: Besides, although the discrimination of our multiple logistic regression model for mortality prediction was satisfactory, we observed significant inadequate fit using the Hosmer–Lemeshow statistic. The large sample size which decrease the power of any goodness of fit test may explain the poor calibration. [40, 41]

13. There is a discrepancy for the linear regression models - in the methods it states scene to hospital time, which is transport time, but in the results the authors discuss total prehospital time. Please clarify the prehospital time interval used

**Response from the authors:**

Thank you for reminding us the mistake.

The dependent variable used in the linear regression in our study was actually total prehospital time and were miswritten in some sentences. We have corrected them.

**Changes made (Please refer to the marked version):**

Page 12, Line 1: To determine the potential factors that may influence total prehospital time, variables that had a p <0.10 on the Mann-Whitney U test were selected for multivariable linear regression analysis, as the dependent variable is continuous, using the forced entry method.

The results are well organized. Some of the introductory demographics text can be removed as it is redundant with Table 1.

**Response from the authors:**

Thank you very much.

We have trimmed the Result section per your advice.

**Changes made (Please refer to the marked version):**

Page 13, Line 9: The sentence “Prehospital management was performed more frequently in patients with 30-day mortality than in those without, with 3.1% placed on supraglottic airway tube and 0.7% on ETT intubation and 12.9% having had an IV or IO line established” was deleted.

The authors present results of a restricted cubic spline regression that was never mentioned in the methods. The authors should include all planned and post-hoc analyses in the methods section.

**Response from the authors:**

Sorry that we did not described it in the Method section

We have added it in the revised manuscript.

**Changes made (Please refer to the marked version):**

Page 11, Line 25: The association of prehospital time and predicted possibilities of favorable functional outcome was presented using the restricted cubic spline regression.

Minor corrections -

"Multivariate" should be changed to "multivariable" regarding the models throughout the text

**Response from the authors:**

Yes, we do mean multivariable and has made changes in the whole manuscript. Thank you for reminding us.

**Changes made (Please refer to the marked version):**

Page 11, Line 19: Multivariable logistic regression was used…

Page 11, Line 21: Variables that had a p <0.10 on the chi-square and Mann-Whitney U tests were selected for multivariable logistic regression analysis…

Page 12, Line 3: …the Mann-Whitney U test were selected for a multivariable linear regression analysis…

Page 14, Line 1: Multivariable logistic regression with overall patient data…

Page 14, Line 24: Multivariable logistic regression showed ….

Page 16, Line 17: … our study included as many confounders in the multivariable logistic regression as possible.

Figure 2 & Legend: Co-variate used in the multivariable logistic regression…

Figure 3 & Legend: Co-variate used in the multivariable logistic regression…

Table 2: TPT was put in the multivariable logistic regression …

Table 3: TPT was put in the multivariable logistic regression…

On the third page of the discussion "scene and play" should be "stay and play"

**Response from the authors:**

Thank you. We have corrected it.

**Changes made (Please refer to the marked version):**

Page 18, Line 16: choosing between “load and go” and “stay and play”

While the authors should be commended for evaluating prehospital time in a large previously understudied population, there are several methodologic concerns and some inherent to the database which limits conclusions that can be drawn from this manuscript.

**Response from the authors:**

Thanks for your insightful comments.

As you mentioned, the inherent bias in a retrospective and observational study will limit the causal inference of the result. It is indeed not appropriate to assertively state a causal inference based on our observational data. Accordingly, we will amend our conclusion to not go beyond what the retrospective cohort design is capable of concluding.

Although with the limitations above, this cross-national, multi-center cohort study still had investigated more than 20 thousand patients. We believe these results should be valuable and informative contribution to the knowledge gap in this area.

**Changes made (Please refer to the marked version):**

Page 4, Line 22: (Abstract) Longer prehospital time was not associated with an increased risk of 30-day mortality but it may increase the risk of poor functional outcomes in injured patients. We supported the concept of “golden hour” for trauma patients during prehospital care in Asia.

Page 23, Line 3: (Conclusion) Although there was no significant association between prehospital time and 30-day mortality in trauma patients, our study suggested rapid transportation for all injured patients because longer prehospital time may be associated with an increased risk of poor functional outcomes.

Page 23, Line 8: (Conclusion) We supported the concept of the “golden hour” for trauma patients during prehospital care in Asia.

-----------------------------------------------------------

Any attachments provided with reviews can be seen via the following link:

__________________________________________________

In compliance with data protection regulations, you may request that we remove your personal registration details at any time. (Use the following URL: https://www.editorialmanager.com/pmedicine/login.asp?a=r). Please contact the publication office if you have any questions.
