# Supplementary material for: Association between prehospital time and outcome of trauma patients in 4 Asian countries: A cross-national, multicenter cohort study
Source: PLoS Med. 2020 Oct 6;17(10):e1003360. doi: 10.1371/journal.pmed.1003360 (PMC7537901; doi:10.1371/journal.pmed.1003360)
Supplement: Attachment — Submitted filename: PMEDICINE-D-20-02427R4 Author Responses.docx [file pmed.1003360.s005.docx]

Dear Dr. WEN-CHU,        

Thank you for resubmitting your manuscript entitled "Association between prehospital time and outcome of trauma patients in Asia: A cross-national, multicenter, cohort study" to PLOS Medicine.  

At this stage, we need to make sure that certain production issues are addressed in order to ensure a smooth handover to our production team, should the manuscript be formally accepted.

Can you please address the following issues as soon as possible? Your paper has been sent back to enable you to make these changes.

EDITORIAL:

1.    Title: Please amend to: “Association between prehospital time and outcome of trauma patients in four Asian countries: A cross-national, multicenter, cohort study” (apologies for asking you to revise this once more, but on further reading we feel this better represents the study)
**Response from the authors:**

Thank you. We had revised the title accordingly

**Changes made (Please refer to the marked version):**

Page 1, Line 1: Association between prehospital time and outcome of trauma patients in four Asian countries: A cross-national, multicenter, cohort study

2.    The Data Availability Statement (DAS) requires revision. Please see the policy at <http://journals.plos.org/plosmedicine/s/data-availability>. For each data source used in your study:
a.    If the data are freely or publicly available, note this and state the location of the data: within the paper, in Supporting Information files, or in a public repository (include the DOI or accession number). Please also provide the minimal data set as described.
b.    If the data are owned by a third party but freely available upon request, please note this and state the owner of the data set and contact information for data requests (web or email address). Note that a study author cannot be the contact person for the data.
c.    If the data are not freely available, please describe briefly the ethical, legal, or contractual restriction that prevents you from sharing it. Please also include an appropriate contact (web or email address) for inquiries (again, this cannot be a study author).
**Response from the authors:**

Thank you.

The data of this study are owned by the PATOS coordination center. Exactly speaking, it is not freely available because of the regulations of PATOS organizations, but an application to get the data is possible, if the application is approved by the PATOS EXCO meeting mainly composing of data-contributing principal investigators from Asia countries.

The contact of the PATOS coordination center is listed below.

Ms. Suhee Agnes KIM, MPH

Laboratory of Emergency Medical Services, Biomedical Research Institute

Seoul National University Hospital | Seoul, South Korea

Tel: +2 2072 4683 | Mobile: +82 10 8572 7715 | http://lems.re.kr/

Email:suheekimsnuh@gmail.com

**Changes made (Please refer to the marked version):**

We had revised it in the submission form.

3.    Abstract:
a.    Please give exact p values over 0.001, otherwise state “p<0.001”.

**Response from the authors:**

Thank you. We had revised it throughout.

**Changes made (Please refer to the marked version):**

Page 3, Line 22: 0.99 (0.92–1.06, p = 0.740), 1.08 (1.00–1.17, p = 0.065), and 1.03 (0.98–1.09, p = 0.236), respectively. However, long prehospital time was detrimental to functional survival. The aORs and 95% CIs of RT, SH, and TPT per 10-minute delay were 1.06 (1.04–1.08, p < 0.001), 1.05 (1.01–1.08, p = 0.007), and 1.06 (1.04–1.08, p < 0.001).

b.    Page 4, line 2: Delete “caused by”

**Response from the authors:**

Thank you. We had revised it accordingly

**Changes made (Please refer to the marked version):**

Page 4, Line 2: “caused by” was deleted

c.    Please correct the data for the following: “The adjusted odds ratios (aORs) and 95% confidence intervals (CIs) per 10 minutes … SH … 1.08 (1.00–1.17, p = 0.07),” – the p value on page 15, line 18 is given as 0.06. Please double check all numerical data in the abstract for accuracy.

**Response from the authors:**

Thank you. We had revised the error.

**Changes made (Please refer to the marked version):**

Page 3, Line 23: (Abstract) 1.08 (1.00–1.17, p = 0.065)

Page 15, Line 19: (Results) 1.08 (1.00–1.17, p = 0.065)

4.    Author Summary:
a.    Page 4, lines 11-12: Please update to “…first proposed early in the 20th century…”

**Response from the authors:**

Thank you. We had revised it accordingly.

**Changes made (Please refer to the marked version):**

Page 4, Line 11: The concept of“golden hour from injury to definitive care” suggesting that critically injured patients should receive definite treatment in 60 minutes first proposed early in the 20th century…

b.    Please add one sentence at the beginning to expand on what the ‘golden hour’ is proposed to be.

**Response from the authors:**

Thank you. We had added the explanation.

**Changes made (Please refer to the marked version):**

Page 4, Line 11: The concept of “golden hour from injury to definitive care” suggesting that critically injured patients should receive definite treatment in 60 minutes first proposed early in the 20th century…

c.    Page 4, line 15: Add “to our knowledge”

**Response from the authors:**

Thank you. We had revised the title accordingly

**Changes made (Please refer to the marked version):**

Page 4, Line 17: There has never been a study to adapt functional status as an outcome measurement for the impact of prehospital time in injured patients to our knowledge.

d.    Page 4, lines 18-20: Update to " This 3-year, cross-national, multi-center cohort study included 24,365 patients from 4 Asian countries (Japan, Korea, Malaysia, and Taiwan).”

**Response from the authors:**

Thank you. We had revised it accordingly

**Changes made (Please refer to the marked version):**

Page 4, Line 21: This 3-year, cross-national, multi-center cohort study included 24,365 patients from 4 Asian countries (Japan, Korea, Malaysia, and Taiwan).

e.    Page 4, lines 23 and 24: Please clarify ‘functional survival’ and ‘functional outcomes’ for a lay reader.

**Response from the authors:**

Thank you. We had added the explanation accordingly

**Changes made (Please refer to the marked version):**

Page 5, Line 2: Poor functional outcome indicates severe disability in daily life or death.

f.    Page 5, line 7: Please clarify ‘unaccounted confounders’ to a lay reader.

**Response from the authors:**

Thank you. We had clarified it.

**Changes made (Please refer to the marked version):**

Page 5, Line 13: …the unaccounted confounders such as quality of prehospital care and in-hospital managements…
g.    Page 5, line 9: Correct typo “Policymakers”

**Response from the authors:**

Thank you. We had revised the typo.

**Changes made (Please refer to the marked version):**

Page 5, Line 15: Policymakers from…

5.    Please remove spaces from within citation callouts and place the callout before punctuation, eg. “…mortality annually worldwide [1,2].” Please keep the space between the text and citation itself.
**Response from the authors:**

Thank you. We had revised it throughout

6.    Page 7, line 24: Please replace “Asia” with the included countries.

**Response from the authors:**

Thank you. We had revised it accordingly

**Changes made (Please refer to the marked version):**

Page 7, Line 23: We conducted a retrospective cohort study of trauma patients admitted to the emergency department (ED) in the included countries…

7.    Page 15, line 24: Please correct the CI to read “0.98–1.09”, as in the Abstract. Please double check all data for accuracy.

**Response from the authors:**

Thank you. We had revised it accordingly. We had also carefully checked the manuscript to ensure all data for accuracy. We found mistakes in “Abstract” and the “Result-characteristics of study objects” section, paragraph 2 and we had revised it. Besides, the P value in the tables and figures and S1 Text were revised into the exact value with the same decimal digits.

**Changes made (Please refer to the marked version):**

Page 3, Line 17: (Abstract) the median age was 45 years old (lower quartile [Q1] – upper quartile [Q3]: 25 – 62)

Page 13, Line 2: Thirty-day mortality was significantly high in older patients (median age 61.0 vs. 45.0, p < 0.001) and in those with non-penetrating injury (99.3% vs. 95.8%, p = 0.004), TBI (non-TBI: 26.4% vs. 66.8%, mixed-TBI: 40.0% vs. 15.8%, isolated TBI = 33.6% vs. 17.3%, p < 0.001), major trauma with an ISS higher than or equal to 16 (66.1% vs. 6.0%, p < 0.001), and RTS lower than 7 (74.6% vs. 4.0%, p < 0.001).

Page 15, Line 24-26: TPT was also not significantly associated with increased odds of mortality (aOR of TPT per 10-minute delay: 1.03, 95% CI 0.98–1.09, p = 0.236)

Table 1, Table 3, Fig2 and S1 Text: P value were revised into the exact value with the same decimal digits.

8.    Results: Please give exact p values over 0.001, otherwise state “p<0.001”.

**Response from the authors:**

Thank you. We had revised it accordingly.

**Changes made (Please refer to the marked version):**

Page 13, Line 2: Thirty-day mortality was significantly high in older patients (median age 61.0 vs. 45.0, p < 0.001) and in those with non-penetrating injury (99.3% vs. 95.8%, p = 0.004), TBI (non-TBI: 26.4% vs. 66.8%, mixed-TBI: 40.0% vs. 15.8%, isolated TBI = 33.6% vs. 17.3%, p < 0.001), major trauma with an ISS higher than or equal to 16 (66.1% vs. 6.0%, p < 0.001), and RTS lower than 7 (74.6% vs. 4.0%, p < 0.001).

Page 15, Line 3: Median RT and SH were all significantly short in patients with RTS lower than 7 (RT: 19.0 vs. 21.0 minutes, p < 0.001; SH: 20.0 vs. 21.0, p = 0.015). Median SH time was significantly longer in patients with ISS higher than or equal to 16 (23.0 vs. 21.0 minutes, p < 0.001). Patients who were placed on prehospital rescue airway tube had a significantly longer median SH (27.0 vs. 21.0 minutes, p = 0.039) than patients who were not. TPT, RT, and SH were all significantly longer if patients received prehospital IV or IO access. Generally, patients with 30-day mortality had a significantly shorter median TPT (41.0 vs. 47.0 minutes, p = 0.006), whereas patients with poor functional outcome had a long median TPT (56.0 vs. 48.0 minutes, p < 0.001).

Page 15, Line 15: Multivariable logistic regression with overall patient data (n = 24,365) revealed that … (adjusted odds ratio [aOR] of RT per 10-minute delay: 0.99, 95% confidence interval [CI] 0.92–1.06, p = 0.740). Long SH may … (aOR of SH per 10-minute delay: 1.08, 95% CI 1.00–1.17, p = 0.065). Other co-variables … (aOR: 1.03, 95% CI 1.03–1.04, p < 0.001), ISS higher or equal than 16 (aOR: 7.14, 95% CI 5.21–9.78, p < 0.001), RTS lower than 7 (aOR: 28.84, 95% CI 20.61–40.37, p < 0.001), rescue airway (aOR: 6.14, 95% CI 1.83–20.60, p = 0.003), and establishment of circulatory access (aOR: 1.61, 95% CI 1.04–2.49, p = 0.033). TPT was also not … (aOR of TPT per 10-minute delay: 1.03, 95% CI 0.98–1.09, p = 0.236) (Table 2). The area under the receiver operating characteristic curve (AUROC) … and the Hosmer–Lemeshow test showed inadequate fit (χ2 = 20.70, p = 0.008)… and the Hosmer–Lemeshow test showed inadequate fit (χ2 = 19.98, p = 0.010).

Page 18, Line 5: The aOR of RT and SH per 10-minute delay were 1.06 (95% CI 1.04–1.08, p < 0.001), 1.05 (95% CI 1.01–1.08, p = 0.007), respectively. A 10-minute delay in TPT was also related to 6% increased odds of poor functional outcome (aOR: 1.06, 95% CI 1.04–1.08, p < 0.001) (Table 3). The AUROC of the multiple logistic regression model for association of RT, SH and functional outcome at discharge was 0.77 (95% CI: 0.76–0.78) and the Hosmer–Lemeshow test showed inadequate fit (χ2 = 37.89, p < 0.001). The AUROC of the multiple logistic regression model for association of TPT and functional outcome at discharge was 0.77 (95% CI: 0.76–0.78) and the Hosmer–Lemeshow test showed inadequate fit (χ2 = 36.55, p < 0.001)

9.    Page 20, lines 11-12: Please clarify “…cohort study of the populations in the countries studied…”

**Response from the authors:**

Thank you. We had revised it accordingly.

**Changes made (Please refer to the marked version):**

Page 20, Line 11: …cohort study of the populations in the countries studied…

10.    Page 21, line 6: Correct to “…data were…”
**Response from the authors:**

Thank you. We had revised it accordingly

**Changes made (Please refer to the marked version):**

Page 21, Line 6: The data were…

FIGURES AND GRAPHICS:

Figure 1 is acceptable for publication.

-Figures 2, 3, and 4 look like they were originally at a lower resolution and will appear blurry or pixelated if published. Text and images should appear crisp and clear. Please note that this problem will not be detected or corrected by the PACE tool, and this will not be fixed by simply increasing the resolution of your Figures in the image editing software. Please carefully review the PLoS Figure Guidelines and provide Figure files that were originally created at 300 pixels/inch (dpi) and at least 6.68cm wide, but less than 19.05cm and under 22.225cm tall. For further information, please see the Figure Guidelines (<http://journals.plos.org/plosmedicine/s/figures>).

**Response from the authors:**

Thank you. Figure 2-4 was revised.

1) Export a PDF from PowerPoint/Word/Excel (or other image source file). On a Mac, click on the 'PDF' button in the printer dialog box and select 'Save as PDF'. On a PC, send the file to print and select 'Adobe PDF' or 'PDFCreator' (it's freeware) from your list of available printers.
If you are using Adobe PDF, then go to Printer Properties, Default Settings and select Press Quality.
If you are using PDFCreator, Go to 'Printer Properties' and then click on 'Advanced' on the bottom right. Under 'Graphic', go to 'Print Quality' and select 600dpi.
2) PHOTOSHOP: When you open up the PDF, make sure that the 'Constrain Proportions' box is checked. Set the 'Resolution' to 300dpi (118.11 pixels/cm) and set the 'Bit Depth' to 8 bit. Use the crop tool (next to the eyedropper) to remove unnecessary white space. Go to - Image - Image Size. Make sure the 'Constrain Proportions' and 'Resample Image” boxes are checked.
To ensure that your Figures publish properly, please delete the alpha channels from each Figure file. In Photoshop, select it in the Channels Palette and click on the trash can icon. When you save the TIFFs make sure that the 'LZW' button is checked and 'Discard Layers and Save a Copy' button is checked.
3) GIMP: When you open up the PDF, set the resolution to 300. Make sure the chain link icon is joined together. Then change the size of your figure according to the pixel specifications mentioned in step 2) above. To ensure that your Figures publish properly, please delete the alpha channels from each Figure file. In GIMP, go to Layers - Transparency - Remove Alpha Channel. When you are done, 'SAVE AS' (or Export if GIMP 2.8). Select File Type - 1st TIFF option. Make sure to use LZW compression.
4) Print out your figures on a desktop printer. Text and graphics should appear clear and crisp with no pixelated or blurred edges.
5) If the resulting figures are still blurry, then your original source images are too low of a resolution. Please increase the resolution of your original source images, and repeat this process.

-Please remove the Figure number/title/caption from Figure 3.
**Response from the authors:**

Thank you. Figure 3 was revised.

-Figure 4 contains font size that is below our recommended range of font sizes. Please note that this problem will not be detected or corrected by the PACE tool. We ask that all type be between 8-12 point (2.8 - 4.2 mm). It is still possible to read 5-6 point type but should only be used sparingly in your figure. For example, it is acceptable for numbers along an axis of a graph or for subscripts and superscripts. 4 point, or smaller, text is not allowed. We recommend that you print figures out to get a better idea of the text legibility.
**Response from the authors:**

Thank you. Figure 4 was revised.

ADDITIONAL PRODUCTION ISSUES:

1. Please ensure that your Ethics Statement is available in the Methods section of your manuscript in its entirety, under the subheading 'Ethics Statement'.
**Response from the authors:**

Thank you. We had revised the Method section with an isolated “Ethics statement” subheading.

**Changes made (Please refer to the marked version):**

Page XX, Line XX: **“Ethics statement”** The PATOS collaboration was approved by the Institutional Review Board of the National Taiwan University Hospital (NTUH). The data were fully anonymized at the time they were accessed by the authors. The study proposal and analysis plan had been approved in PATOS Taipei meeting, Nov 7, 2015. The original study proposal is shown in Table A in S1 Text. The PATOS trauma database is characterized by an EMS-based registry. The initial proposal (2015) of current study planned to analyze the association between “the golden hour (i.e. injury time to definite care in hospital)” and trauma patient outcomes. However, when the PATOS phase 1 data were released in 2019, we found that there was an inadequate sample size of patients with ISS≥16, therefore the analysis plan was revised to test the association between prehospital timeliness and outcome.

2. Please replace the page numbers in your STROBE statement with paragraph numbers per section (e.g. "Methods, paragraph 1") since the page numbers of the final published paper may be different from the page numbers in the current manuscript.
**Response from the authors:**

Thank you. We had already revised it in the last revision and we will ensure it.

3. Please note that your submission must refer to specific files using our naming convention (S1 Fig, S1 Table, S1 Text, S1 Data, etc.) Please update all in-text citations and supporting information file legends/captions accordingly (do not use underscores in the manuscript text).
**Response from the authors:**

Thank you. We had confirmed it.

Please log in at <https://www.editorialmanager.com/pmedicine/>. Click the 'Revisions Sent Back to Author' link to access your submission.

You can then make the changes requested above by clicking 'Edit Submission' from the list of Action Links. Once you have completed your edits please 'View Submission' and then click 'Approve Submission' to resubmit.   

If you have any questions please feel free to contact us at [plosmedicine@plos.org](mailto:plosmedicine@plos.org).          

Best regards,

Jessica Hyde
Publications Assistant
PLOS Medicine
[plosmedicine@plos.org](mailto:plosmedicine@plos.org)
